# Chronic and Transient Loneliness in Spanish Adults: Prevalence, Risk Factors, and Their Impact on Depression, Anxiety, and Substance Use Disorders

**DOI:** 10.1192/j.eurpsy.2025.1478

**Published:** 2025-08-26

**Authors:** Í. Gete-Alejandro, J. Domènech-Abella

**Affiliations:** 1 Recerca, Parc Sanitari Sant Joan de Déu, Sant Boi de Llobregat, Spain

## Abstract

**Introduction:**

Chronic and transient loneliness are two clinically distinct conditions that have different impacts on mental health. However, there is limited information regarding the prevalence of chronic and transient loneliness, and their associated risk factors.

**Objectives:**

This study aims to (1) assess the prevalence of chronic and transient loneliness among Spanish adults, (2) identify and compare the risk factors associated with both forms of loneliness, (3) examine how perceptions of loneliness contribute to its persistence, and (4) evaluate the effects of chronic versus transient loneliness on depression, anxiety, and substance use disorders.

**Methods:**

A total of 1,503 Spanish adults were interviewed by phone from May to June 2024. Loneliness was measured with a direct question, and the three-item loneliness scale from the University of California, Los Angeles, was used for the sensitivity analyses. Chronic loneliness was defined as reported loneliness both two years ago and currently, while transient loneliness was defined as reported loneliness only two years ago. The CAGE Adapted to Include Drugs (CAGE-AID) questionnaire was used to assess substance use disorder within the previous month, anxiety symptoms were measured using the GAD-2, and depressive symptoms were assessed using the PHQ-2. Logistic regression models were constructed to examine sociodemographic and behavioral risk factors for chronic and transient loneliness, as well as the impact of loneliness perceptions on the likelihood of chronicity and the association of both types of loneliness with mental disorders.

**Results:**

11.4% of the sample reported chronic loneliness, while 9.5% reported transient loneliness. Risk factors associated with chronic loneliness included being a younger adult, female, living alone or with roommates, having low education level, and poor health status. In contrast, the risk factors for transient loneliness included being single or separated and frequent use of social media. Poor social support was a risk factor for both types of loneliness, though it had a significantly stronger impact on chronic loneliness (OR = 10.1, 95% CI: 5.5–18.4) compared to transient loneliness (OR = 2.2, 95% CI: 1.2–3.8). Feelings of emptiness (OR = 2.7, 95% CI: 1.4–5.4) and perceiving loneliness as insurmountable (OR = 2.6, 95% CI: 1.4–4.6) were risk factors for chronicity among individuals feeling loneliness. Sensitivity analyses showed similar results to the main findings. Only chronic loneliness was longitudinally associated with higher odds of depression (OR = 5.0, 95% CI: 3.4–7.4), anxiety (OR = 3.1, 95% CI: 1.3–4.1), and substance use disorder (OR = 3.6, 95% CI: 1.9–6.6).

**Conclusions:**

Chronic loneliness can be identified through its risk factors and the perceptions of those experiencing it, highlighting the importance of addressing these issues to improve mental health outcomes.

**Disclosure of Interest:**

None Declared

